# Comparative study of uranium and thorium metal ion adsorption by gum ghatti grafted poly(acrylamide) copolymer composites

**DOI:** 10.1039/c9ra08212c

**Published:** 2019-12-13

**Authors:** Gauri Shelar-Lohar, Satyawati Joshi

**Affiliations:** Department of Chemistry, Savitribai Phule Pune University Pune Maharashtra India id-ssjoshi@chem.unipune.ac.in jayapune@gmail.com; Department of Chemistry, Fergusson College Shivajinagar Pune Maharashtra India

## Abstract

Uranium and thorium ions were selectively removed from aqueous solution using synthesized gum ghatti grafted poly(acrylamide) gum-*g*-poly(AAm) composite. A gamma radiation induced free radical copolymerization technique was used to synthesize the copolymer composite of gum-*g*-poly(AAm). Fourier transform infrared spectroscopy (FTIR), thermogravimetric analysis (TG), X-ray diffraction (XRD) and field emission scanning electron microscopy (FESEM) were used to characterize the graft copolymer gum-*g*-poly(AAm). The adsorption of uranium ions and thorium ions using the gum-*g*-poly(AAm) copolymer composites has been investigated in batch mode. The adsorptive characteristics were investigated by varying the pH, concentration and time for both ions. The adsorption method depends on the pH of each metal ion, and the highest adsorption percentage was achieved at pH 6.0. The adsorption statistics were justified by isotherm, kinetic and thermodynamic models. The Langmuir adsorption model was revealed to be the best fitted monolayer arrangement, with a maximum adsorption capacity of 367.65 mg g^−1^ for the uranium ions and 125.95 mg g^−1^ for the thorium ions. The adsorption of metal ions occurred by the ion exchange process, which was specified through the rate controlling step with a best-fitted pseudo-second order kinetic rate model. Thermodynamic analysis shows that the Δ*H* and Δ*S* values for the uranium ions and thorium ions were positive. The negative Δ*G* values decreased with an increase in temperature, suggesting that the metal ion adsorption process was endothermic and spontaneous in behaviour.

## Abstract 2

Uranium and thorium ions were selectively removed from aqueous solution using synthesized gum ghatti grafted poly(acrylamide) copolymer composite.

## Graphical abstract

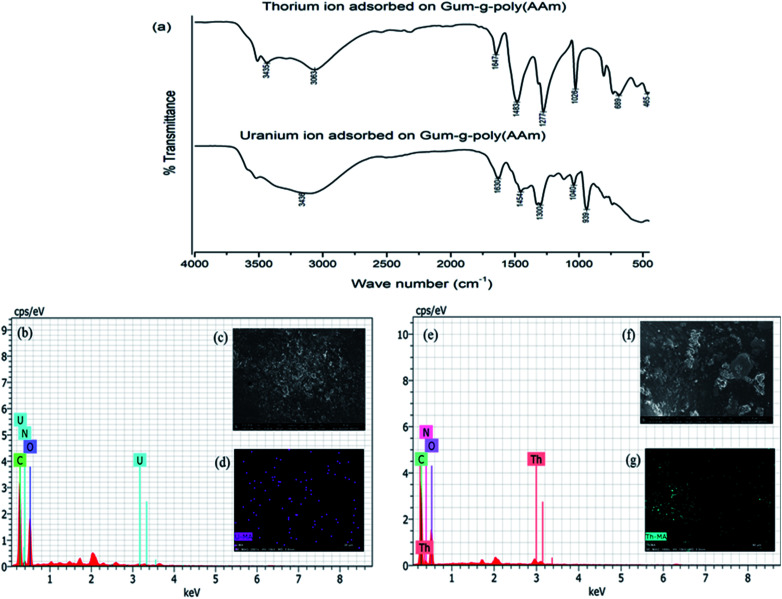

## 1. Introduction

Uranium and thorium are essential nuclear fuel elements; the interests for these components have expanded tremendously and also have found potential in the fields of super conductors, rechargeable batteries, propelled earthenware production and fibre optics.^1^ The groundwater and some sources of surface water have been contaminated by the radioactive uranium and thorium ions, which cause harm to the ecosystem, environment and human health.^2^ At different pH ranges, uranium and thorium in solution exist in a complexed form. For example, Th^4+^ is the significant species in an aqueous medium at pH > 2. Under an acidic condition, it may be hydrolysed to Th(OH)_2_^2+^, Th(OH)_3_^+^, Th(OH)^3+^ and Th_2_(OH)_4_^4+^. With an increase in the pH value to an alkaline condition, it will precipitate as Th(OH)_4_.^3^ Uranium exists in the dimer form (UO_2_)_2_(OH)_2_^2+^ in the lower acidic range from pH 2 to 5. In the pH range of 5–10, the predominant species of uranium is found in the form of UO_2_(OH)_2_. In the pH range of 7–9, (UO_2_)_8_O_2_(OH)_12_·12H_2_O (schoepite) precipitates.^4^

Subsequently, it becomes imperative to reduce the concentrations of these radioactive elements from industrial effluents before they are released to nature. Numerous methods have been utilized for radioactive element removal such as adsorption, ion exchange, biodegradation, photocatalysis, flocculation and coagulation.^5^ Adsorption as a selective separation method for the efficient recovery of uranium and thorium is most desirable for environment protection and for nuclear energy.^6^ The efforts are diverted more on optimizing the surface properties. The affinity of uranium and thorium ions can be enhanced by surface modification with novel functional groups.^7,8^

Numerous studies have been carried out on various low-cost adsorbents such as activated sludge,^9^ seaweed,^10^ starch,^11^ cellulose^12^ and gum.^13^ Polysaccharide-based polymers have been mostly and effectively used for heavy metal ion adsorption from wastewater.^14–16^ Gum ghatti is biocompatible, and is naturally and abundantly available. Gum ghatti is acquired as exudates of the *Anogeissus latifolia* tree. *Anogeissus latifolia* is a type of small-to-average sized tree local to India, and is found in Western Ghats. They have numerous benefits over conventional adsorbents. The grafted polymer composites of gum ghatti with vinyl monomer possess good mechanical and physical properties over the ungrafted composites.^17,18^

For graft copolymer synthesis, acrylamide is a potential monomer. Acrylamide is water-soluble and can furnish the grafted copolymer chains with an amide group. The average molecular weight of gum ghatti is 8.94 × 10^7^ g mol^−1^. The main composition of gum ghatti is sugar in the form of l-arabinose, d-galactose, d-mannose, d-xylose and d-glucuronic acid in a 48 : 29 : 10 : 5 : 10 molar proportion. It has alternate 4-*O*-substituted and 2-*O*-substituted α-d-mannopyranose segments with chains of 1 → 6 connected β-d-galactopyranose segments as the side chains, which are most persistently the single l-arabinofuranose unit.^19–21^

Recently, a few studies on the removal of radioactive waste with different adsorbents were performed. Impregnated cellulosic beads synthesized by the chemical precipitation method were employed for the removal of toxic U(vi) ions.^22^ Using the plasma initiation method, gelatin-modified attapulgite was synthesized for the uptake of uranium.^23^ The superabsorbent grafted copolymer composite of poly(methacrylic acid) with cellulose/bentonite synthesised by the chemical initiation method (using potassium per sulphate as the initiator) was used for the recovery of thorium(iv).^24^

The present work reports the synthesis of gum ghatti grafted copolymer with acrylonitrile by the gamma irradiation induced method. The gamma irradiation route has several advantages over other synthesis routes. No chemical initiator is required to initiate the polymerization, and there are no side products. In addition, one can control the reaction by altering the radiation dose. The synthesis was carried out under ambient conditions. FTIR, TGA, FESEM, XRD and BET analysis were used to characterize the synthesized gum-*g*-poly(AAm) composite. This study is focused on the adsorption behaviour of synthesized gum-*g*-poly(AAm) for the selective adsorption of uranium and thorium ions. The relationships between the adsorbents' behaviour and their competence, as well as the adsorption mechanism, are evaluated by equilibrium adsorption isotherms, and by kinetic and thermodynamic assessment.

## 2. Experimental

### 2.1 Materials

All reagents used for synthesis were pure. Gum ghatti (AR grade) was purchased from Hi-media. Acrylamide (AR grade, 99.8%), methanol (AR grade, 99.5%), hydrochloric acid (AR grade, 99.9%), sodium hydroxide (AR grade, 99.5%) and acetone (AR grade, 99.5%) were obtained from Merck Chemicals, India. Uranyl nitrate and thorium nitrate were purchased from Sigma.

### 2.2 Synthesis of graft copolymer of gum ghatti with acrylamide (gum-*g*-poly(AAm))

1.0 gram of gum ghatti was dissolved in 20.0 mL distilled water at 75 ± 2 °C under nitrogen atmosphere to prepare a 5.0% gelatinous slurry of gum ghatti, and cooled at room temperature. 3.0 grams of acrylamide (1 : 3 w/w ratio of gum ghatti to monomer) was added to the gelatinous slurry, and the stirring was continued for the next 30 min to form a homogeneous mixture with continuous nitrogen purging. The prepared mixture was irradiated under a ^60^Co gamma source having a dose rate of 1.0 kGy min^−1^ for 20 min (*i.e.*, for a total dose of 20.0 kGy). The irradiated sample was precipitated by adding methanol, and then filtered and dried at 50 °C in an oven. Further, the grafted copolymer was purified by acetone to remove any homopolymer using Soxhlet extraction.^25^ The polymer composite was then dried at 50 °C to a constant weight and characterized by different techniques.

### 2.3 Characterization

The FTIR spectra of gum ghatti and gum-*g*-poly(AAm) were confirmed on a Fourier transform infrared spectrophotometer in the range of 400 to 4000 cm^−1^ on a Shimadzu FTIR 8400s spectrometer. The thermogravimetric study of gum ghatti and gum-*g*-poly(AAm) were obtained using a PerkinElmer (Pyris Diamond TG/DTA, USAA) thermal analyzer at a heating rate of 10 °C min^−1^. The surface morphology of the gum ghatti and grafting appearance of gum-*g*-poly(AAm) were examined using field emission scanning electron microscopy (FESEM, FEI, Nova NanoSEM 450).

### 2.4 Adsorption study

The uranium and thorium ion concentrations were calculated using Arsenazo III at 650 nm and 660 nm, respectively, by a UV-Vis spectrophotometer.^26^ The solution pH was adjusted with 0.1 N NaOH and 0.1 N HCl. In a 25.0 mL metal ion solution, 0.05 g of gum-*g*-poly(AAm) was added. To evaluate the kinetics and isotherm parameters, the adsorption study was optimized as a function of pH, agitation time and the initial concentration of the metal ions. The adsorption percentage (%Ad) and adsorption capacity (*q*_e_) of both metal ions were determined from the following equations:1
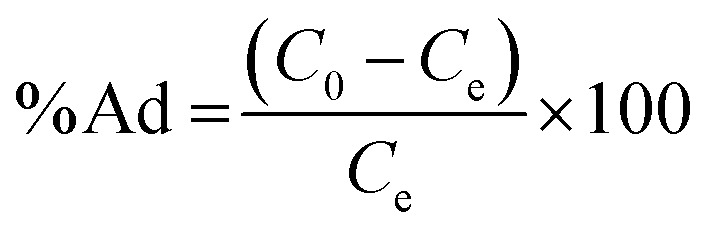
2
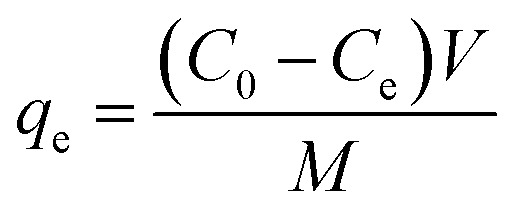
where *C*_0_ and *C*_e_ are the initial and equilibrium concentration of the metal ions, respectively, *V* is the volume of the metal ion solution, and *M* is the weight of the adsorbent.

### 2.5 Desorption and reusability

The desorption of the uranium and thorium metal ions was carried out in different eluents, such as 0.1 N HCl, H_2_SO_4_, HNO_3_ and CH_3_COOH. The composites of the adsorbed metal ions gum-*g*-poly(AAm) (0.05 g) were treated with a 25.0 mL acid solution, and the amounts of desorbed metal ions were determined by a UV-Vis spectrophotometer. Furthermore, to test the reusability of gum-*g*-poly(AAm), the adsorption–desorption experiment was repeated three times. After the desorption experiment, the gum-*g*-poly(AAm) composites were washed with distilled water to remove excess acid and used for further adsorption cycles.

The desorption percent was calculated by the following equation:3
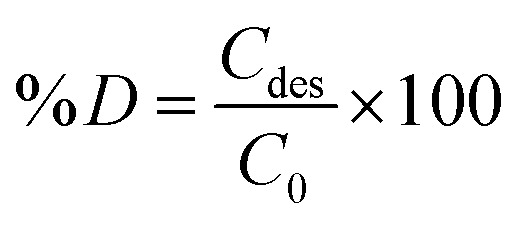


## 3. Results and discussion

### 3.1 Characterization

**Fig. 1:**
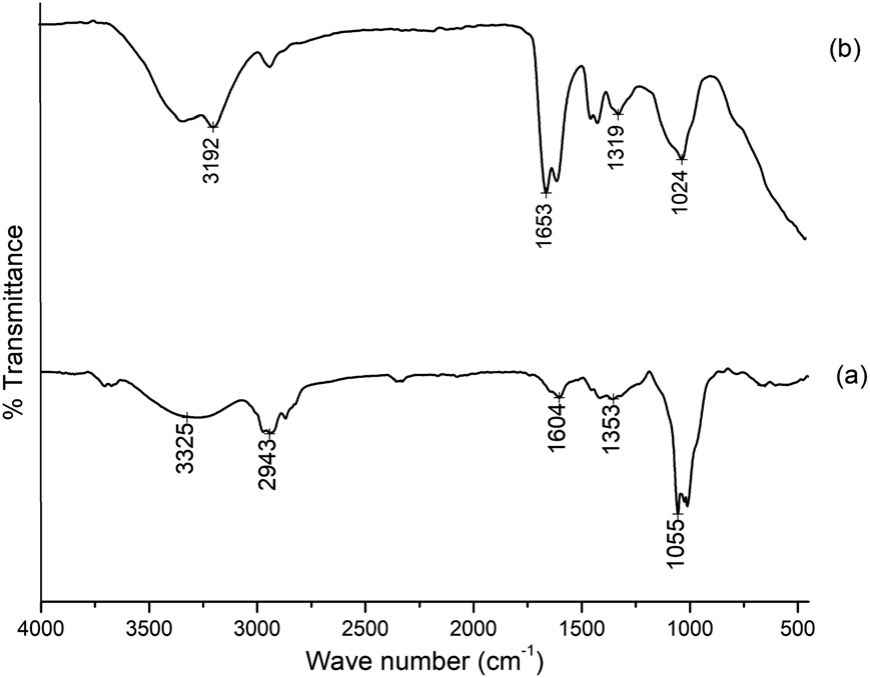
FTIR of (a) gum ghatti and (b) gum-*g*-poly(AAm).

The FTIR spectra of gum ghatti and the gum-*g*-poly(AAm) composite are shown in Fig. 1. The typical peaks of gum ghatti were observed at 3256 cm^−1^, 2943 cm^−1^ and 1620 cm^−1^, and are assigned to the –OH stretching mode of polysaccharide, and the stretching vibrations of the –CH group and –C=O group, respectively. In the spectrum of gum-*g*-poly(AAm), the characteristic band at 1456 cm^−1^ is attributed to the amide stretching vibration. In addition, the band at 1622 cm^−1^ contributing to the –C=O stretching mode indicated the presence of the acrylamide group. An additional band at 3525 cm^−1^ that is overlapping the broad band shows an increase in intensity due to OH and NH stretching from gum ghatti and AAm, which confirms that the grafting reaction of gum ghatti with acrylamide was completed successfully.

**Fig. 2:**
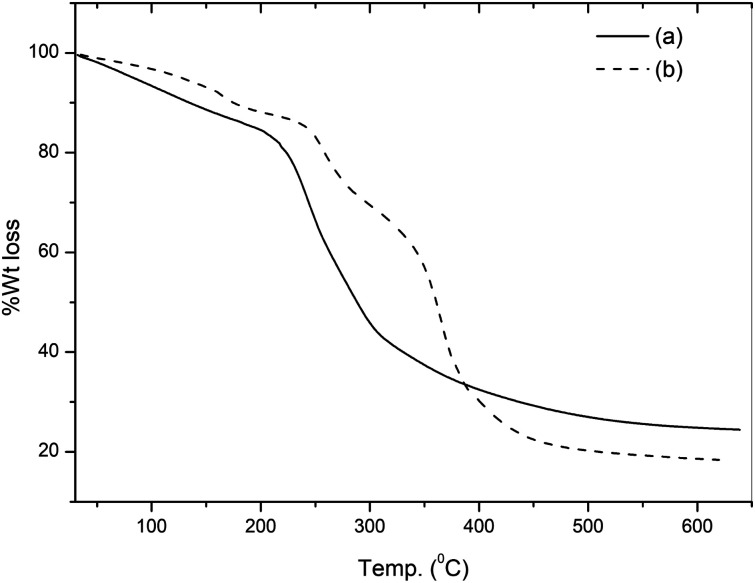
TGA of (a) gum ghatti and (b) gum-*g*-poly(AAm).

The TG curves of pure gum ghatti and the gum-*g*-poly(AAm) composite are illustrated in Fig. 2. The TG curve of gum ghatti showed a two-step degradation. The weight loss of gum ghatti comprises the consequent stages: an initial 10.6% weight loss observed in the temperature range 55 to 170 °C is due to dehydration; the second weight loss starts from 170 to 420 °C with a 55.9% weight loss for the complete thermal decomposition of gum ghatti. Conversely, gum-*g*-poly(AAm) shows three distinct weight losses: the first weight loss between 55 to 180 °C with a 9.2% loss in weight is due to the loss of moisture or the degradation of the ungrafted gum chain; the second stage ranging from 215 to 310 °C with a 19.5% weight loss is due to the depolymerisation of the backbone polymeric chain, and the last degradation commenced from 310 to 510 °C with a 37.8% weight loss is attributed to the complete degradation of gum-*g*-poly(AAm). It also confirms that the decomposition temperature of gum-*g*-poly(AAm) in the grafted polymer is much higher than that of pure gum ghatti. The total weight losses of gum ghatti and gum-*g*-poly(AAm) are 74.2% and 66.5%, respectively. Thus, the thermal stability of the backbone polymer (gum ghatti) was enhanced noticeably by grafting with AAm.

**Fig. 3:**
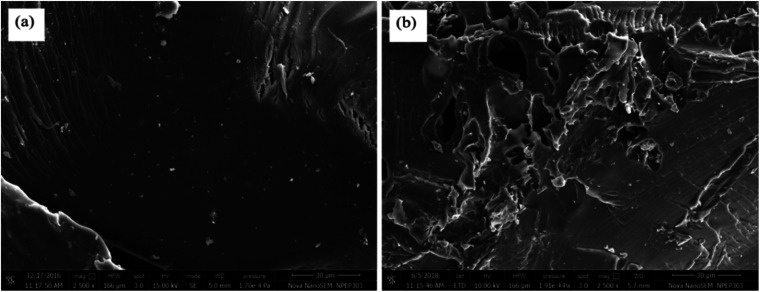
FESEM of (a) gum ghatti and (b) gum-*g*-poly(AAm).

Gum ghatti and gum-*g*-poly(AAm) composite were analysed by FESEM microscopy, and exposed substantial information in regards to their surface morphology. As it appears in the FESEM micrograph (Fig. 3), gum ghatti exhibits a less uneven surface. By grafting the monomer acrylamide with gum ghatti, the surface becomes irregular and crosslinked, and contains more pores with enhanced monomeric units and reduced backbone contents. Thus, these data reveal that the grafting procedure was done successfully.

### 3.2 Adsorption of metal ions

#### 3.2.1 Effect of pH on adsorption

**Fig. 4:**
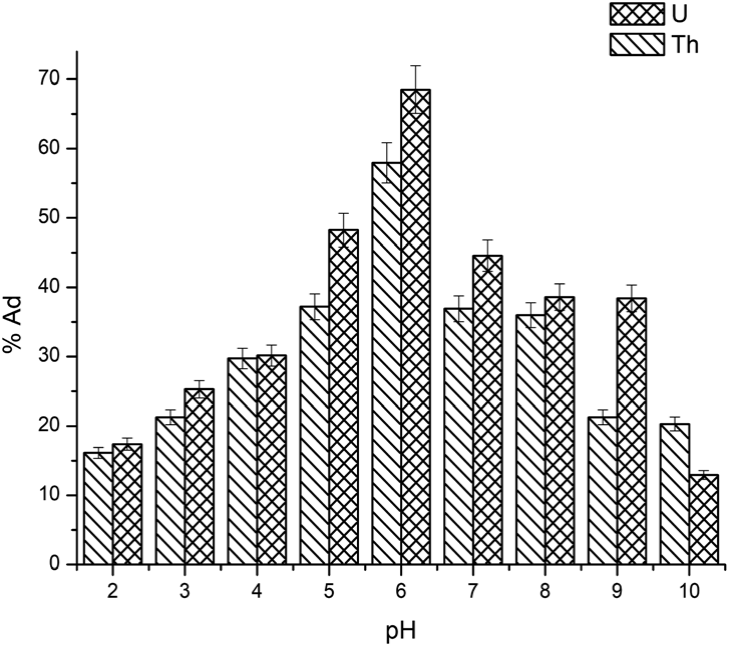
Effect of pH on the adsorption of uranium and thorium ions.

In all performed studies, the pH of the solution plays a vital role, which influences the adsorption capacity and both the metal ions considerably in solution. The effect of the solution pH on the adsorption of metal ions was investigated using 0.05 g gum-*g*-poly(AAm) in 25 mL of 100 mg L^−1^ concentration of metal ions solution separately. The desired pH of the solution was adjusted with 0.1 M HCl or 0.1 M NaOH with a range of pH values from 3 to 9. Fig. 4 illustrates that for the uranium and thorium ions, the percent adsorption was increased from pH 3.0–6.0 and further decreased with an increase in pH. At the lower pH range of 3.0–5.0, uranium exists in the dimer form. The hydrolysed species of the thorium ions showed a slight increase in the percent adsorption, and the adsorption process may be limited due to the ionization of the surface functional groups and the surface charge. At pH 6.0, the percent adsorption was high. At this pH, UO_2_^2+^ and Th^4+^ are the key species in aqueous medium, which is useful for the complexation response, and thereby enhancing the chelating ability of gum-*g*-poly(AAm). With an increase in pH > 6.0 (*i.e.*, in alkaline condition), the percent adsorption was decreased, which may be due to the formation of stable complexes of uranium and thorium as (UO_2_)_8_O_2_(OH)_12_ and Th(OH)_4_ precipitates, respectively.^27,28^ Consequently, the optimum pH during the adsorption process was maintained at 6.0 for further experiments.

#### 3.2.2 Adsorption isotherm

**Fig. 5:**
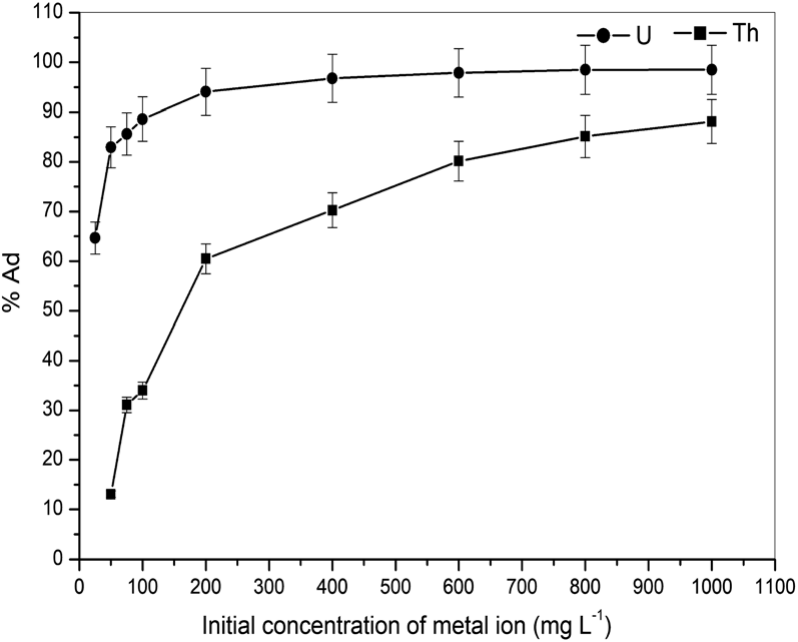
Effect of initial concentration on the adsorption of uranium and thorium ions.

To determine the interface and adsorption behaviour of the adsorbent to the adsorbate, the appropriate mathematical equations will describe it in detail at equilibrium and at a constant temperature. The limitation for the implication of the adsorption process will depend on the relationship between the solution and the adsorbed phase at equilibrium, and on the specific adsorbent–adsorbate systems equilibrium data. The adsorption behaviour of gum-*g*-poly(AAm) was determined as the effect of initial metal ion concentrations. The equilibrium uptake of the metal ions was estimated by treating 0.05 g of adsorbate with 50 to 1000 mg L^−1^ concentration of metal ion solution. It was observed that *q*_e_ increases progressively with an increase in *C*_e_, and gradually reaches the maximum adsorption capacity for the uranium and thorium ions as the initial concentration was varied from 25 to 1000 mg L^−1^ (as shown in Fig. 5).

**Fig. 6:**
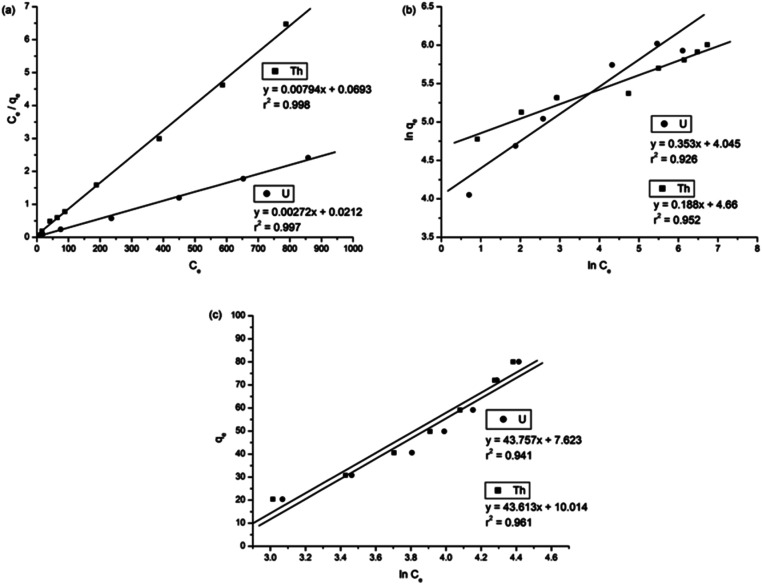
(a) Langmuir adsorption isotherm, (b) Freundlich adsorption isotherm, and (c) Temkin adsorption isotherm.

To understand the adsorption behaviour, different isotherm models such as the Langmuir, Freundlich, and Temkin isotherms have been employed to ensure the adsorption performance.^29^Fig. 6 shows the adsorption behaviour of all adsorption isotherm models. The Langmuir model implies that the metal ions are distributed from the aqueous to the solid phase at equilibrium, and also showed the related monolayer arrangement of the adsorbate onto the adsorbent. This is restricted to the identical sites of adsorption for constant adsorption energy, and there is no interaction between the adsorbed molecules and the adjacent binding sites of the adsorbate. In addition, the Freundlich isotherm signifies that the adsorption process is predominantly heterogeneous. According to the Freundlich isotherm theory, the ratio of the amount of solute adsorbed onto a given mass of adsorbent to the concentration of the solute is not constant at different concentrations in the solution. A smaller value of the Freundlich constant implies that the adsorption of the adsorbate onto the adsorbent is easy.^30^ The Temkin adsorption isotherm model and adsorbent–adsorbate interactions provide information on the impacts of interaction between the adsorbent and adsorbate on the adsorption process and heat of adsorption (Δ*H*_ads_), signifying the adsorption process as a physical adsorption or chemical adsorption. This model assumes that the adsorbent has uniform binding energy sites, and the heat of adsorption of all molecules in a layer decreases linearly due to the adsorbate–adsorbent interactions. The mathematical linear formulae of all isotherm models are given in Table 1.

**Table 1:** Isotherm and kinetic parameters for uranium and thorium ion adsorption by gum-*g*-poly(AAm)

Adsorption isotherm models	Linear equation forms	Parameter description		Values for uranium ions	Values for thorium ions
Langmuir isotherm model^31^	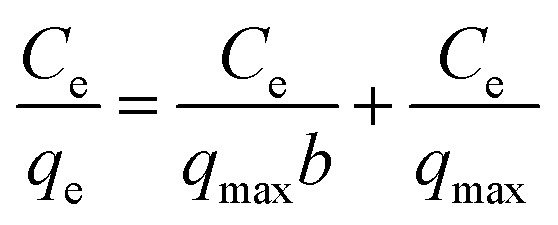 , 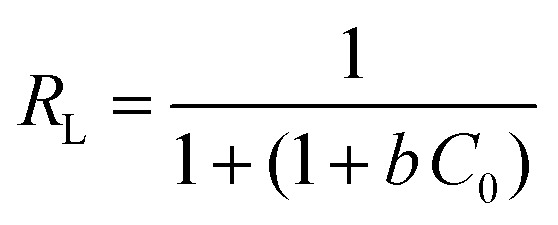	*q*_max_ = adsorption capacity at equilibrium, (mg g^−1^)		367.65	125.95
		*b* = bonding energy of adsorption, (L mg^−1^)		1.253 × 10^−1^	1.145 ×10^−1^
		*R*_L_ = equilibrium parameter		0.138	0.148
		*r* ^2^		0.997	0.998
Freundlich isotherm model^32^	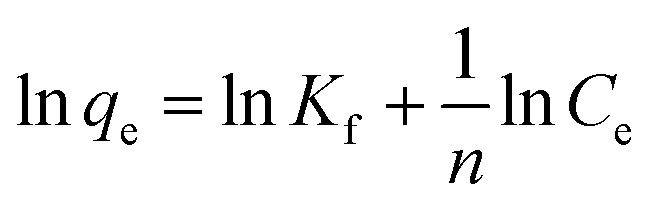	*K*_f_ = strength of the adsorptive bond, (L g^−1^)		57.111	95.583
		*n* = the adsorption intensity, (g L^−1^)		2.833	5.139
		*r* ^2^		0.926	0.952
Temkin isotherm model^33^	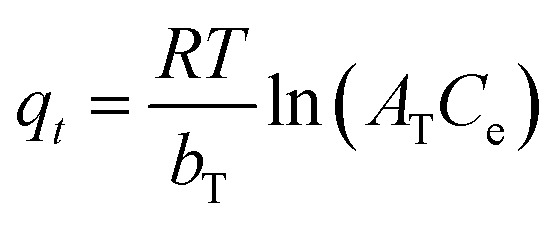	*b* = Temkin constant related to heat of the adsorption, (J mol^−1^)		20.470	26.986
		*a* = equilibrium binding constant, (L g^−1^)		0.168	0.154
		*r* ^2^		0.984	0.971
Pseudo-first order kinetic model^35^	ln(*q*_e_ − *q*_*t*_) = ln *q*_e_ − *k*_1_*t*	*k*_1_ = rate constant (min^−1^)		1.38 ×10 ^−2^	2.04 ×10 ^−2^
		*q*_e_ = adsorption capacity, (mg g^−1^)		210.81	358.252
		*r* ^2^		0.968	0.988
Pseudo-second-order kinetic model^36^	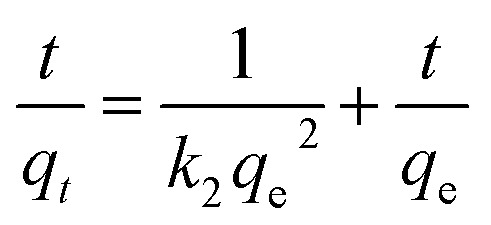	*k*_2_ = rate constant, (g mg^−1^ min^−1^)		1.095 × 10 ^−4^	3.436 × 10 ^−4^
		*q*_e_ = adsorption capacity, (mg g^−1^)		353.35	172.41
		*r* ^2^		0.998	0.993
Weber Morris model^37^	*q*_*t*_ = *Kt*^0.5^ + *C*	*K* = intra-particle diffusion rate constant, (mg g^−1^ min^1/2^)	Step 1	27.528	0.262
			Step 2	12.642	0.087
			Step 3	0.592	0.019
		*C* = intercept, (mg g^−1^)	Step 1	72.384	3.845
			Step 2	218.227	5.759
			Step 3	319.084	6.888
		*r* ^2^	Step 1	0.986	0.996
			Step 2	0.945	0.936
			Step 3	0.998	0.979

With *r*^2^ of 0.997 and 0.998 for the uranium and thorium ions, respectively, the Langmuir data is better fitted than the other two adsorption isotherm models. As indicated by the Langmuir isotherm adsorption model, the adsorption takes place by monolayer adsorption. The adsorption limit was reached to maximum evaluation with 367.65 mg g^−1^ uranium ions and 125.95 mg g^−1^ thorium ions. The significance of the Langmuir adsorption isotherm was verified by the estimations of *R*_L_, which is in the range of 0–1 (Table 1) for the adsorption. The equilibrium binding constant (*a*) (L g^−1^) and the Temkin constant (*b*) (related to the heat of the adsorption (J mol^−1^)) were determined for both metal ions from the Temkin plot, which showed that the adsorption process followed physisorption. Furthermore, a greater value of *q*_m_ for the uranium ions than thorium ions was due to the higher binding capacity of the uranium ion with the gum-*g*-poly(AAm) composite. The thermal motion may interfere with the adsorption of ions and being selective for adsorption. The adsorption capacities for both metal ions are different, and this may be due to a difference in the ionic radius. With increasing ionic radius, the steric crowding on the adsorption surface will also increase; thus, a saturation limit of adsorption is rapidly attained.

From these adsorption isotherm studies, it was observed that the adsorption capacity values for gum-*g*-poly(AAm) for the uptake of uranium ions and thorium ions were different. The adsorption of metal ions depends on various characteristic properties of the metal ions, such as the ionic radius, hydration energy, electronegativity and solubility. The ionic radii, hydration energy and electronegativity values for the uranium ion are 0.97 Å, −3958 kJ mol^−1^ and 1.38 eV; whereas for the thorium ions, the values are 1.19 Å, −3332 kJ mol^−1^ and 1.30 eV, respectively. With decreasing cationic radii, the hydration energy will increase since it ideally adsorbs the metal ions faster. In the case of the uranium ion, it is available in the form of uranyl ions (UO_2_^2+^) in aqueous medium. Due to the presence of oxygen, it may show an increase in the ionic size, which leads to a decrease in the hydration energy compared to the uranium ions. Under an aqueous condition, thorium is available in the Th^4+^ form. Therefore, in the present study, it was observed that the adsorption capacity of the uranium ion is more. This difference in the adsorption capacity is not only related to the metal ion properties, but is also due to the different properties of the adsorbent, such as the presence of the functional groups, the surface area and surface morphology.

#### 3.2.3 Kinetic study

Adsorption kinetics is essential for the assessment of the adsorption competence. In the first place, the impact of the agitation time and the straight-line behaviour of various models are intended to recognize the adsorption rate. The effect of the agitation time on the adsorption of the uranium and thorium ions was studied for 30 to 180 min using a 0.05 g amount of gum-*g*-poly(AAm) in 100 mg L^−1^ metal ion solution at pH 6.

**Fig. 7:**
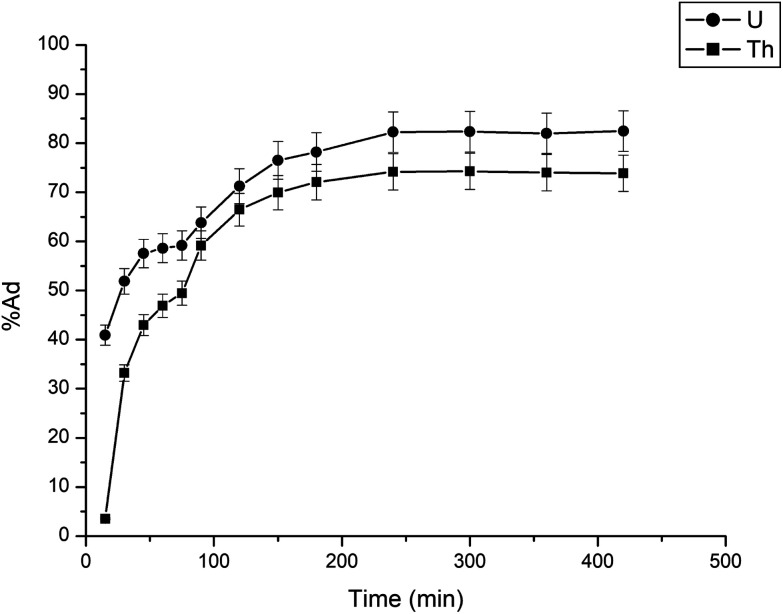
Effect of agitation time for the adsorption of uranium and thorium ions.

The adsorption of uranium and thorium ions was calculated and is illustrated in Fig. 7. The adsorption rates extended rapidly within the initial time because of the significant available adsorption sites and enhanced concentration gradient. Due to the occupied adsorption sites and decreasing concentration gradient, the adsorption rate decreased and reached equilibrium.^34^ Equilibrium was achieved within 2 h for uranium ion uptake and 3 h for thorium ion uptake by the gum-*g*-poly(AAm) composites. Four adsorption kinetic models (pseudo-first order, pseudo-second order, intraparticle diffusion, and Elovich models) were studied and are shown in Fig. 7. Their mathematical expressions are shown in Table 1.

**Fig. 8:**
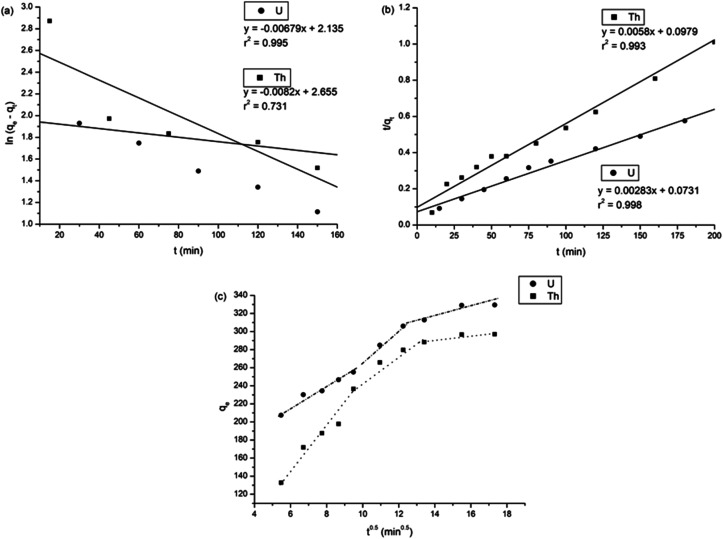
Kinetics model (a) pseudo-first order, (b) pseudo-second order, and (c) Weber Morris model.

Fig. 8 shows all kinetics models for uranium and thorium adsorption. According to the regression coefficients (*r*^2^) of the straight fitting, the pseudo-second order model was revealed best fitted for the uranium ions and thorium ions with *r*^2^ = 0.998 and *r*^2^ = 0.996, respectively. The pseudo-second order adsorption gives information about the interaction between the adsorbate and adsorbent. This model describes the rate of adsorption of the number of active/binding sites occupied on the adsorbent surface as being proportional to the square of the number of available sites on the adsorbent at equilibrium. The Weber–Morris model illustrates that, in many adsorption cases, the metal ion uptake varies proportionally with *t*^1/2^. As seen from Fig. 8(c), for both metal ions, the intraparticle diffusion plots show three-step processes. It describes the boundary layer diffusion of the adsorbate to the adsorbent. The Weber–Morris plots for both metal ions do not pass through the origin, which shows that intraparticle diffusion processes are controlled to some extent by the boundary layer diffusion. The first step indicates that the diffusion of the metal ions occurs through the solution to the external surface of the gum-*g*-poly(AAm), or may be through the boundary layer diffusion of the adsorbent molecule. The second step diffusion describes the diffusion of the adsorbent into the mesopores of the adsorbate particles. From the third step diffusion, it was observed that diffusion occurs through the micropores of the adsorbate, which exhibits the lowest slope relating to the rate-limiting step in the adsorption process. This may be influenced by various factors such as the size of the adsorbate molecule, concentration of the metal ions, affinity of the metal ions to adsorb, diffusion coefficient of the metal ions within the bulk phase, and the pore size distribution to the adsorbate.

#### 3.2.4 Thermodynamics

For the adsorption of the metal ions, the temperature is an important parameter to understanding the thermodynamics of the adsorption process. The kinetic energy and thermodynamic properties are directly related to each other. From this relation, it is easy to understand the diffusion of the adsorbate onto the adsorbent surface at different stages. To know whether the adsorption process is spontaneous or not, thermodynamic consideration should be taken into account. For these circumstances, thermodynamic parameters such as the free energy change (Δ*G*), enthalpy (Δ*H*) and entropy (Δ*S*) of adsorption were determined based on the Van't Hoff plot.^38^ The thermodynamic study was performed from 303 to 333 K. The plot of ln *K*_d_*vs.* T^−1^ can be used to determine thermodynamic parameters, such as enthalpy change (Δ*H*) in kJ mol^−1^ and entropy (Δ*S*) in J mol^−1^ K^−1^:4
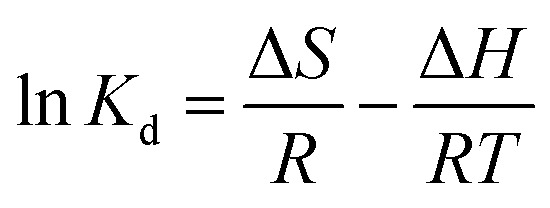
where *R* = ideal gas constant (8.314 J mol^−1^ K^−1^), *T* = temperature (K), and *K*_d_ = distribution coefficient (*K*_d_). The distribution coefficient can be calculated as follows:5
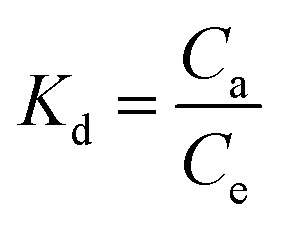
where, *C*_a_ = the equilibrium adsorbate concentration on the adsorbent (mg L^−1^), and *C*_e_ = the equilibrium adsorbate concentration in solution (mg L^−1^).

The change in the Gibbs free energy was calculated from the equation:

6 Δ*G* = Δ*H* − *T*Δ*S*

The obtained values of Δ*H* and Δ*S* calculated from the plot of ln *K*_d_*vs.* T^−1^ are listed in Table 2. The positive values for Δ*H* and Δ*S* indicate that the adsorption process is endothermic and there will be a small amendment within the structural appearance of the adsorbate surface, increasing the randomness at the solid–solution interface. Negative Δ*G* values were decreased with increasing temperature, which suggest a spontaneous adsorption of both ions.

**Table 2:** Thermodynamic parameters for uranium and thorium ion adsorption by Gum-*g*-poly(AAm)

Parameter	Δ*H* (kJ mol^−1^)	Δ*S* (kJ mol^−1^ K^−1^)	Δ*G* (kJ mol^−1^)			
			303 K	313 K	323 K	333 K
Values for uranium ions	85.48	0.284	−0.568	−3.408	−6.248	−9.088
Values for thorium ions	112.19	0.377	−2.041	−5.811	−9.581	−13.351

### 3.3 Adsorption mechanism with spectroscopic confirmation

**Fig. 9:**
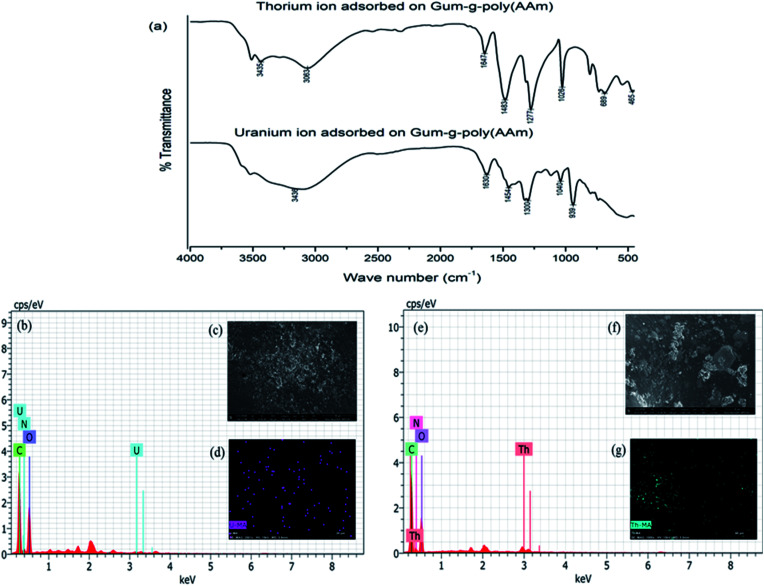
(a) FTIR of uranium and thorium ions adsorbed on gum-*g*-poly(AAm); (b), (c) and(d) EDX, FESEM and elemental analysis of uranium ion adsorbed on gum-*g*-poly(AAm); (e), (f) and (g) EDX, FESEM and elemental analysis of thorium ions adsorbed on gum-*g*-poly(AAm).

The FTIR spectra of the gum-*g*-poly(AAm) composite for the adsorption of the uranium and thorium ions are shown in Fig. 9(a). Compared with the before and after adsorption of the uranium and thorium ions, both exhibited weaker peaks at 1652 cm^−1^ (C=N vibrating mode). The peak at 3332 cm^−1^ (–OH vibration) of the gum-*g*-poly(AAm) composite was red-shifted to 3436 cm^−1^ and 3435 cm^−1^ due to uranium and thorium ion adsorption, respectively. These indicated that the N atoms of the acrylamide groups and the O atoms from the gum ghatti backbone chain may be involved in the adsorption process.^39^ Therefore, if the –NH_2_ of acrylamide and –OH of the gum ghatti functional groups participated in the coordination, then the metal ions may be trapped in the monomer and backbone chains. In addition to IR spectroscopy, FESEM, EDX and elemental analysis also supported the confirmation of uranium and thorium ion adsorption onto the gum-*g*-poly(AAm) composite. The EDX spectra (Fig. 9(b) and (e)) show a distinct peak for uranium and thorium ions after adsorption onto gum-*g*-poly(AAm). The FESEM images exhibited a change in the surface morphology due to the adsorption of the metal ions onto the gum-*g*-poly(AAm) surface. All pores of the adsorbate were occupied by the metal ion adsorption, which was spotted by elemental mapping. It has been observed that the surface modification of the adsorbate with –NH_2_ leads to the enhancement in the adsorption rate of the metal ions.

### 3.4 Desorption and reusability study

**Fig. 10:**
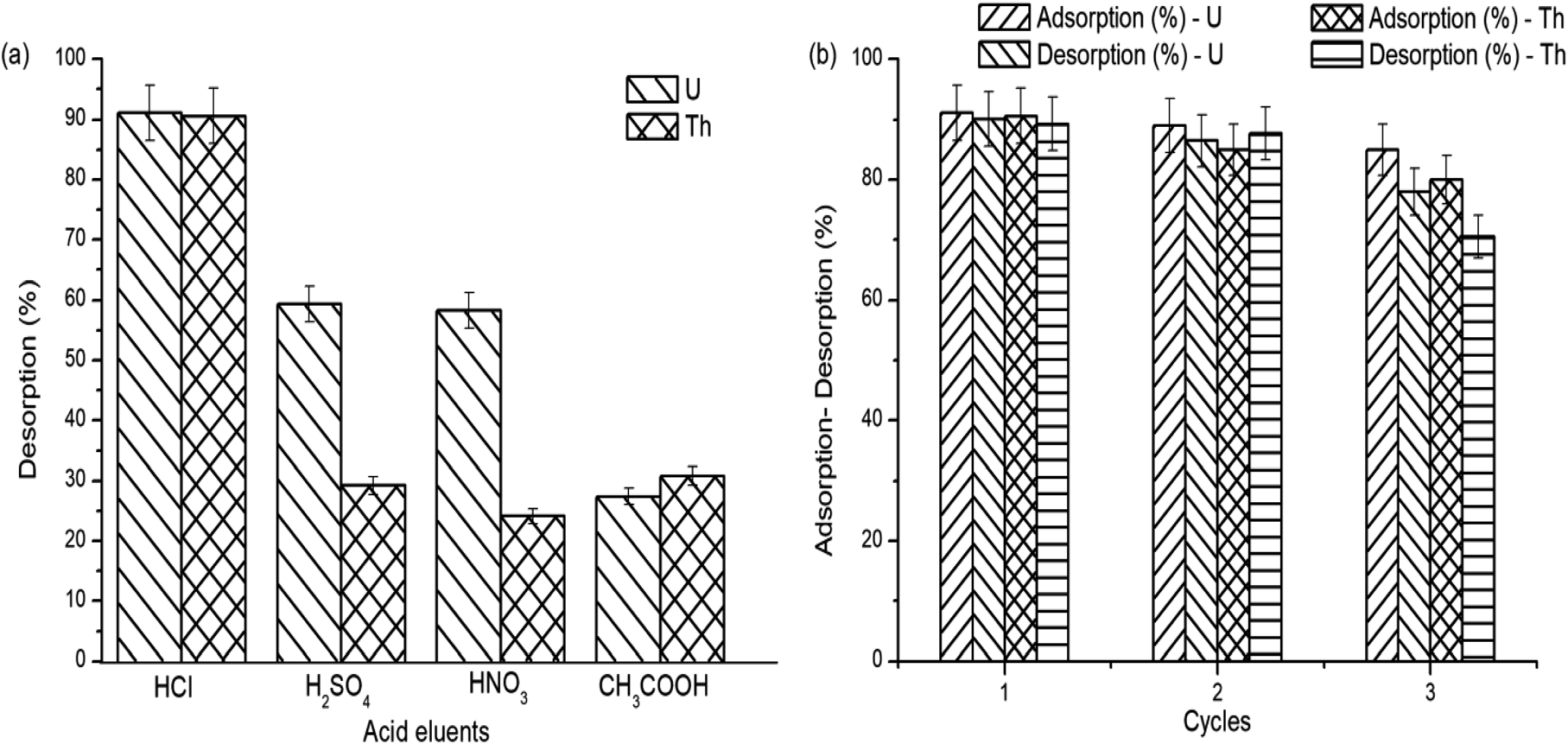
(a) Desorption study and (b) regeneration study.

The regeneration and the reusability capacity of the grafted copolymer are important for a valuable development. The desorption of the adsorbed uranium and thorium ions on gum-*g*-poly(AAm) was studied using various acid eluents. The maximum desorption capacity was obtained in 0.1 N HCl, as shown in Fig. 10(a).

To further assess the reusability, 0.1 N HCl was used as a desorption eluent. After three adsorption and desorption cycles (Fig. 10(b)), the adsorption capacity of gum-*g*-poly(AAm) was decreased from 91.1% to 85.0% and 89.8% to 80% for the removal of uranium and thorium ions, respectively. This shows that gum-*g*-poly(AAm) can be used effectively after the regeneration of metal ions.

### 3.5 Comparison with other adsorbent

In order to justify the ability of the gum-*g*-poly(AAm) composite as an adsorbent, its sorption potential should be compared with different adsorbents, which are given in Table 3. The adsorption capacity of the gum-*g*-poly(AAm) composite was higher than that of the other adsorbents.

**Table 3:** Comparison of maximum adsorption capacity of uranium and thorium ions using various adsorbents

Metal ions	Adsorbents	Maximum adsorption capacity (mg g^−1^)
Uranium ions	Gum-*g*-poly(AAm) composite (present work)	367.65
	Layered double oxide/carbon dot nanocomposites^40^	354.2
	MAO-chitosan beads^41^	117.65
	Polyaniline (PANI) modified bentonite^7^	14.1
	TMP-*g*-AO^8^	35.37
Thorium ions	Gum-*g*-poly(AAm) composite (present work)	125.95
	PVA/Fe_3_O_4_/SiO_2_/APTES nanohybrid adsorbent^42^	62.5
	Tannin modified poly(glycidyl methacrylate) grafted zirconium oxide densified cellulose (TMPGZDC)^43^	96.69

## 4. Conclusion

Gum-*g*-poly(AAm) composite copolymer was employed for the effective removal of uranium and thorium ions at pH 6. The adsorption kinetics and isotherm showed the best fitted data with the pseudo-second-order model and Langmuir isotherm, respectively, for both metal ions. The adsorption capacity for uranium ions (367.65 mg g^−1^) was found to be greater than that of the thorium ions (125.95 mg g^−1^). The spontaneity of the adsorption process was indicated by the thermodynamic parameters. The obtained Δ*H* and Δ*S* values were positive, indicating the endothermic nature of the adsorption and increase in the degree of freedom, whereas the negative Δ*G* values showed that the adsorption process was spontaneous. The overall studies show that the gum-*g*-poly(AAm) composite possesses a competent absorption capacity for removing heavy metal ions, and is an excellent sorbent carrier for the environmental remediation study.

## Conflicts of interest

There are no conflicts to declare.

## Supplementary Material
